# Loss of zinc‐finger protein 143 contributes to tumour progression by interleukin‐8‐CXCR axis in colon cancer

**DOI:** 10.1111/jcmm.14290

**Published:** 2019-04-01

**Authors:** Vikas Verma, A Rome Paek, Beom-Kyu Choi, Eun Kyung Hong, Hye Jin You

**Affiliations:** ^1^ Translational Research Branch, Div. of Translational Science Goyang Gyeonggi South Korea; ^2^ Biomedicine Production Branch Research Institute, National Cancer Center Goyang Gyeonggi South Korea; ^3^ Department of Pathology National Cancer Center Hospital Goyang Gyeonggi South Korea; ^4^ Department of Cancer Biomedical Science, National Cancer Center Graduate School of Cancer Science and Policy National Cancer Center Goyang Gyeonggi South Korea

**Keywords:** cytokine, interleukin‐8, macrophage, STAT3, ZNF143

## Abstract

Several studies have shown that expression of zinc‐finger protein 143 (ZNF143) is closely related to tumour progression including colon cancer. However, it remains unclear how ZNF143 expression is related to tumour progression within the tumour microenvironment. Here, we investigated whether ZNF143 expression affects the tumour microenvironment and tumour progression by screening molecules secreted by colon cancer cells stably expressing short‐hairpin RNAs against ZNF143 or control RNAs. We observed that secretion of interleukin (IL)‐8 was increased when ZNF143 expression was reduced in two colon cancer cell lines. The mRNA and protein levels of IL‐8 were increased in cells following ZNF143 knockdown, and this effect was reversed when ZNF143 expression was restored. The Janus tyrosine kinase/signal transducer and activator of transcription (JAK/STAT) and extracellular signal‐regulated kinase pathways were also shown to contribute to IL‐8 expression in ZNF143‐knockdown cells. The expression levels of ZNF143 and IL‐8 were inversely correlated with three‐dimensionally grown spheroids and colon cancer tissues. THP‐1 cells were differentiated when cells were incubated with condition media from colon cancer cell with less ZNF143, drastically. Loss of ZNF143 may contribute to the development of colon cancer by regulating intracellular and intercellular signalling for cell plasticity and the tumour microenvironment respectively.

## 1 INTRODUCTION

Colorectal cancer (CRC) is the third most common cancer and the second most common cause of cancer‐related deaths worldwide among both males and females. CRC represents a serious health concern as high rates of metastasis and recurrence result in survival rates less than 15%.^1^ The progression of cancer cells to a metastatic state involves many molecular changes; however, the critical changes driving metastasis remain unclear.^2^, ^3^

Evidence has suggested that metastasis is initiated by the spreading of tumour cells, which is facilitated by the formation of supportive metastatic microenvironments, referred to as pre‐metastatic niches or tumour microenvironments, which develop prior to primary tumour cell dissemination.^4^

The tumour microenvironments are composed of tumour cells, tumour stromal cells, the extracellular matrix, oxygen levels and so on. Stromal cells including macrophages, endothelial cells and fibroblasts contribute to tumour‐supportive environments by expressing growth factors, cytokines and chemokines, which are the main mediators for communication between tumours and stromal cells.^5^, ^6^ In colon cancers, macrophages were shown to interact and contribute to colon cancer progression,^7^ supporting the role of macrophage within tumour microenvironments through intercellular communications. There are still many questions how macrophages are activated and differentiated into tumour‐associated macrophages (TAMs) for tumour progression and metastasis. However, it is clear that the communication between stromal cells including macrophages and cancer cells is important for regulating tumour survival and growth within tumour microenvironment.

The communication within tumour microenvironment is characterized by the presence of cytokines, chemokines and receptors. Chemokines mediate the accumulation of immunocompetent cells and help in shaping a tumour‐promoting or ‐suppressive microenvironment. Although the tumour microenvironment is being increasingly recognized as a key factor in cancer aggressiveness, the underlying mechanisms remain controversial.^8^

Cancer cells up‐regulate the expression of numerous cytokines by expressing receptors that benefit their own survival. Cytokines are soluble proteins that play an important role in inflammation as well as in the initiation and promotion of carcinogenesis.^9^ Interleukin (IL)‐8, which is also known as CXCL8, is one of the most significantly up‐regulated chemokines in CRC, indicating its potential diagnostic value.^10^ IL‐8 acts by binding to the cell surface receptors CXCR1/2, and the downstream signalling pathway contributes to tumour growth and invasion, inducing CRC cell proliferation and migration.^11^ IL‐8 signalling has been associated with activation of the classic mitogen‐activated protein kinase (MAPK) signalling cascade, with downstream phosphorylation of extracellular signal‐regulated kinase (ERK)‐1/2 in cancer cells.^12^ Furthermore, increased phosphorylation of signal transducer and activator of transcription (STAT)‐3 has been detected in patients with colorectal carcinoma and was associated with metastasis and poor prognosis.^13^

Zinc‐finger protein 143 (ZNF143), a ubiquitously expressed transcriptional activator that belongs to the Kruppel family of zinc‐finger proteins, has been implicated in the transcriptional regulation of genes associated with the cell cycle and DNA replication.^14^ Accumulating evidence has suggested that ZNF143 is involved in a variety of cellular and pathogenic processes.^15^, ^16^, ^17^, ^18^, ^19^, ^20^ The role of ZNF143 as a transcriptional regulator has been studied in various cancers, such as lung adenocarcinoma, as well as in colon, prostate, breast, gastric cancers and leukaemia.^20^, ^21^, ^22^, ^23^ Recently, we found that the expression of ZNF143 was reduced in invasive ductal carcinoma tissue compared to normal epithelial breast tissue, suggesting a role in cancer cell motility and invasion.^24^

In the present study, we demonstrate the role of ZNF143 in regulating IL‐8 expression via the crosstalk of MAPK and Janus tyrosine kinase (JAK)/STAT signalling, associated with intercellular communication with immune cells which maintains the tumour microenvironments for enhanced CRC cell invasiveness.

## 2 MATERIALS AND METHODS

### 2.1 Materials

DMEM and defined foetal bovine serum (FBS) were obtained from GIBCO (Grand island, NY). Matrigel was obtained from Corning (Bedford, MA). Mouse monoclonal antibodies against β‐actin, IL‐8 and ZNF143 were obtained from Santa Cruz Biotechnology, Inc (Santa Cruz, CA). Rabbit monoclonal antibodies against *p*‐p38/p‐38, *p*‐ERK/ERK, *p*‐JNK/JNK, *p‐*p65/p65‐NFκB and STAT3 families were obtained from Cell Signaling Technology, Inc (Beverly, MA). Horseradish peroxidase‐conjugated antimouse and anti‐rabbit antibodies were purchased from Cell Signaling Technology. Short‐hairpin (sh) RNA‐lentiviral particles against human ZNF143, and the control were purchased from Santa Cruz Biotechnology, Inc. Specific inhibitors, Cucurbitacin I hydrate (JSI 124) and Stattic (for JAK/STAT pathway), were obtained from Sigma‐Aldrich. PD98059 (for MEK/ERK pathway), BAY 11‐7085 (for NFκB pathway) and SB225002 (for CXCR1/2 inhibitor) were obtained from Calbiochem^®^ Inc (Merck KGaA, Germany). Recombinant human tumour necrosis factor (TNF)α was purchased from Calbiochem^®^ Inc (Merck KGaA, Germany). BD cytometric bead array (CBA) Human Soluble Protein Master Buffer kit for IL‐8 measurement was purchased from BD, USA. Human ZNF143 cDNA (GenBank Accession No. NM_003442) was amplified and cloned for expression (pFLAG‐CMV2‐hZNF143FL) as described previously.^20^ Primers used for PCR and cloning are available on request. Fragments obtained by PCR and subcloning were confirmed by DNA sequencing. Proteome Profiler™ Human Cytokine Array Kit, Panel A was purchased from R&D Systems (Minneapolis, MN).

### 2.2 Cell culture

The human colon carcinoma cell lines HCT116 and HT29 were obtained from the American Type Culture Collection (Manassas, VA) and THP‐1, human monocytes, were from Korean cell bank (KCLB, 40202, South Korea). All cells were authenticated by short‐tandem repeat PCR method in 2017 (HCT116 and HT29) and in 2018 (THP‐1). HCT116 cells were maintained as monolayers in DMEM. HT29 cells were maintained in McCoy 5A media. THP‐1 cells were maintained as suspension culture in RPMI 1640. All maintenance media were supplemented with 10% heat‐inactivated FBS. All cells were grown at 37°C in a humidified 5% CO_2_ atmosphere.

### 2.3 Short‐hairpin RNA‐mediated silencing of human ZNF143 in colon cancer cells

To achieve stable lentivirus‐mediated expression of short hairpin RNA (shRNA) specific for the gene encoding ZNF143, HCT116 and HT29 cells were grown for 24 hours, incubated with 5 mg/mL polybrene for 1 hour, and infected with the lentiviral vector (approximately 1 molar ratio of infection) as described previously.^25^, ^26^

### 2.4 Cytokine profile assessment

To screen for cytokine expression and secretion, we used the Proteome Profiler Human Cytokine Array Kit, Panel A (Research & Diagnostic Systems, Inc, Minneapolis, MN). The supernatants isolated from cells (700 µL) were subjected to profiling according to the manufacturer's instructions. The cytokines present were detected by exposing the membrane to X‐ray film, which was subsequently developed. The mean luminescence was normalized to reference spots from the same membrane following background correction.

### 2.5 Flow cytometry‐CBA assay

Cells (5 × 10^5^) were seeded for 24 hours followed by the treatment. Supernatants were collected to measure IL‐8 secretion, whereas cell lysates were used to estimate synthesis of IL‐8. Supernatants and cell lysates were processed using the CBA Human Soluble Protein Master Buffer kit according to the manufacturer's instructions (BD Biosciences, Franklin Lakes, NJ). Briefly, 50 µL of supernatant was mixed with 50 µL of capture beads, with 1 µL of IL‐8 capture beads for each reaction, and incubated for 1 hour, followed by the addition of 50 µL of detection reagent and incubation for 2 hours at room temperature. Samples were washed, resuspended in wash buffer and analysed using the FACSVerse™ flow cytometer (BD Biosciences) equipped with 488 and 633 nm lasers.

### 2.6 Isolation of RNA, reverse transcription‐PCR and real‐time PCR

Cells (5 × 10^5^) were grown in 6‐well plates for 24 hours and harvested. Total RNA was extracted with the RNeasy kit (Qiagen, Valencia, CA). RNA was quantified, and samples (5 µg) were reverse‐transcribed at 42°C for 60 minutes in 20 µL buffer (10 mmol/L Tris, pH 8.3, 50 mmol/L KCl, 5 mmol/L MgCl_2_ and 1 mmol/L dNTP) in the presence of a random hexamer primer. Hot‐start PCR was performed to increase the specificity of amplification. The PCR products were subjected to electrophoresis on 1.5% (w/v) agarose gels, and the resulting bands were visualized with ethidium bromide and photographed using the GelDoc program (Bio‐Rad, Chicago, IL). For real‐time PCR quantification, reactions were performed with LightCycler FastStart DNA Master SYBR Green I (Roche Diagnostics Corp., Indianapolis, IN) as follows: 95°C for 10 minutes, followed by 45 cycles of 95°C for 15 seconds and 58‐62°C for 30 seconds. Relative fold expression was calculated using the 2^−ΔΔCt^ method. Glyceraldehyde 3‐phosphate dehydrogenase mRNA was used as an endogenous control.^27^ All primer sequences are provided in Table S1.

### 2.7 Immunoblotting

Cells were harvested in lysis buffer containing a protease and phosphatase inhibitor cocktail and quantified using the bicinchoninic assay according to the manufacturer's instructions (Pierce, Thermo Fisher Scientific Ltd). Protein samples were heated at 95°C for 5 minutes and separated by SDS‐PAGE using 8‐15% acrylamide gels, followed by transfer to polyvinylidene difluoride membranes. The membranes were blocked for 1 hour in Tris‐buffered saline with 0.01% Tween‐20 (TBST) with 3% bovine serum albumin (BSA), after which they were incubated overnight with primary antibody in TBST with 2% BSA, followed by incubation with horseradish peroxidase‐conjugated antimouse or ‐rabbit antibody. The blots were developed with an enhanced chemiluminescence kit (West‐ZOL plus, Western Blot Detection System; Intron Biotechnology, Inc, Daejeon, South Korea), and quantification of band intensity on XAR‐5 film (Eastman Kodak Co., Rochester, NY) was measured with Quantity One software (Bio‐Rad).

### 2.8 Cell fractionation

Cells were lysed and separated into cytosolic, membrane, nuclear and cytoskeletal fractions using the Qproteome Cell Compartment kit (Qiagen) according to the manufacturer's instructions. Briefly, harvested cell pellets were resuspended in ice‐cold extraction buffers CE1, CE2, CE3 and CE4, followed by different incubation times and centrifugation, ultimately resulting in the separation of cytosolic, membrane, nuclear and cytoskeletal proteins. The soluble protein fractions underwent acetone precipitation for downstream applications. Proteins were quantified, and immunoblotting was performed.

### 2.9 Immunofluorescence staining

Cells were fixed in 4% paraformaldehyde and permeabilized in 0.25% Triton X‐100 in phosphate‐buffered saline (PBST). Cells were incubated with 1% BSA diluted in PBST for 30 minutes with gentle rocking to block non‐specific binding. Finally, cells were immunostained with the primary antibody (phosphorylated STAT3 at Tyr^705^ [*p*‐STAT3^Tyr705^]) and secondary antibody in PBST with 1% BSA. Cells were mounted with anti‐fade Vectashield mounting medium (Vector Laboratories, Inc, Burlingame, CA) and visualized under a confocal microscope.

### 2.10 Three‐dimensional cell cultures

Matrigel was mixed with serum‐free media to a final concentration of 3 mg/mL. Hundred‐microlitre of the medium was added to 96‐well culture plate and allowed to solidify in CO_2_ incubator for overnight. Next day, harvested cells were counted, and 100 cells were seeded onto Matrigel followed by 100 μL of growth media over the Matrigel. Cells were grown into spheroids for 14 days. Further these spheroids were harvested and counted for reseeding and RNA isolation.

### 2.11 Treatment of monocytes with colon cancer cell conditioned medium (CM)

Colon cancer cells (5 × 10^5^) were seeded in 6‐well plates. Next day, 2 mL of fresh media was replaced and kept for 24 hours to get the conditioned medium (CM) from cancer cells. Cancer cell CM was collected and centrifuged at 3000 rpm for 10 minutes. THP‐1 cells were grown in RPMI 1640 medium supplemented with 10% FBS and 50 μmol/L β‐mercaptoethanol. For the experiment, THP‐1 cells (2 × 10^5^/mL) was either treated with dimethyl sulfoxide (DMSO) (DMSO, Sigma‐Aldrich), 100 nmol/L phorbol 12‐myrisatate13‐acetate (PMA, Sigma‐Aldrich) or 50% of CM from cancer cells for 24 hours in 12‐well plates.

### 2.12 Immunohistochemistry and scoring

ZNF143, IL‐8 and *p*‐STAT3^Tyr705^ staining of tissue arrays (US Biomax, Inc, Rockville, MD; http://www.biomax.us/, slide BC05002b) was conducted by the National Cancer Center Animal Sciences Branch. Colon tissue in the tissue microarrays was stained with haematoxylin and mouse anti‐ZNF143, IL‐8 (1:500) or *p*‐STAT3^Tyr705^ (1:100) antibodies and detected with 3,3′‐diaminobenzidine. The stained tissues on the tissue microarray were digitized at 20× magnification using the Aperio AT Turbo whole slide scanner (Leica Biosystems, Buffalo Grove, IL) equipped with a clinical grade RGB camera. Expression was quantified as a percentage of ZNF143/IL‐8/*p*‐STAT3^Tyr705^‐positive nuclei (0‐3, negative to strong) relative to the total number of haematoxylin‐positive nuclei per tumour. Images from the slide not stained with primary antibody were used as a negative control.

### 2.13 Statistical analysis

All data are expressed as percentages of the control and shown as means ± standard error (S.E.). Statistical comparisons between groups were performed with Student's *t* tests. Values of *P* < 0.05 were considered significant.

## 3 RESULTS

### 3.1 IL‐8 expression is increased following ZNF143 knockdown in colon cancer cells

**Figure 1:**
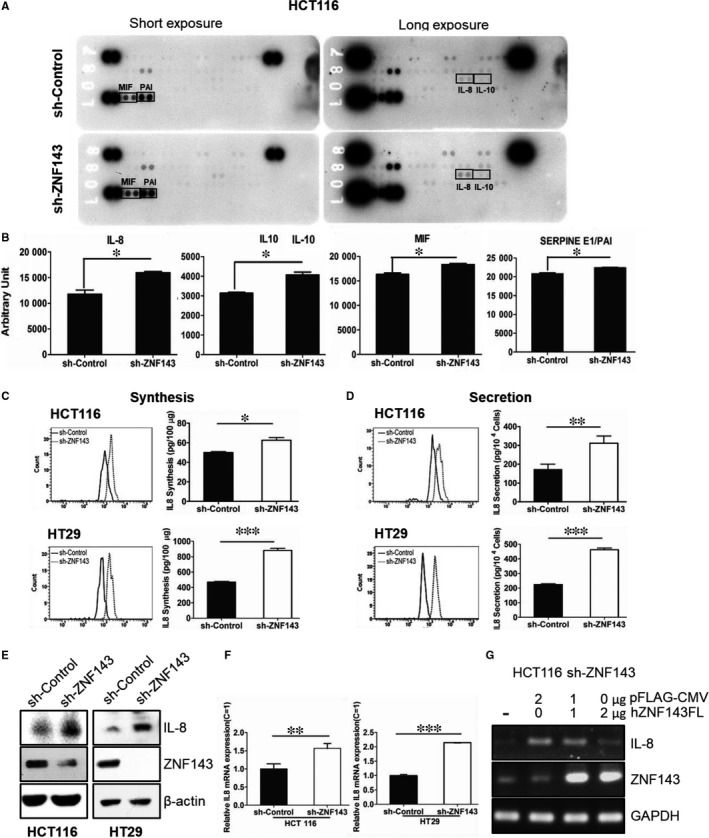
Knockdown of ZNF143 increases the expression of IL‐8 in colon cancer cells. (A, B) The supernatants isolated from cells were subjected to profiling (A) and the intensities of cytokines were analysed (B). Cells were grown for 24 hours, harvested and analysed for IL‐8 synthesis (C) and secretion (D) by FACS as described in the Materials and Methods section. (E, F) Cells were harvested and analysed for mRNA and protein expression of IL‐8 by real‐time polymerase chain reaction (PCR). (F) and immunoblotting (E) respectively. (G) Cells were transfected with pFLAG‐CMV or plasmid encoding the full open reading frame of the human ZNF143 gene and harvested for reverse transcription (RT)‐PCR analysis. Data are expressed as means ± S.E. of at least three independent experiments. Statistical significance was assessed using unpaired Student's *t* tests (*P < 0.02; **P < 0.05; and ***P < 0.0001). Results shown are representative of at least three independent experiments

We examined the effect of ZNF143 knockdown on the cytokine profile of the colon cancer cell line HCT116. After 24 hours of growth, media from control and ZNF143‐silenced HCT116 cells were obtained and subjected to proteomic profiling (Figure 1A,B). IL‐8, IL‐10 and macrophage inhibitory factor (MIF) were found to be significantly increased in the supernatants of HCT116 sh‐ZNF143 cells (Figure 1A), whereas the levels of IL‐6 were low, and SERPINE1/plasminogen activator inhibitor (PAI) levels were only marginally altered. MIF is a pleiotropic cytokine that is overexpressed in a number of solid tumours, including breast, prostate and colon cancers.^28^ PAI‐1 initiates several events that promote cancer; clinically, increased levels of PAI‐1 in colon cancer epithelia are associated with increased metastasis.^29^ IL‐8, an important pro‐inflammatory chemokine, has been shown to modulate endothelial cell migration and promote angiogenesis, tumourigenesis and metastasis.^30^, ^31^

**Figure 2:**
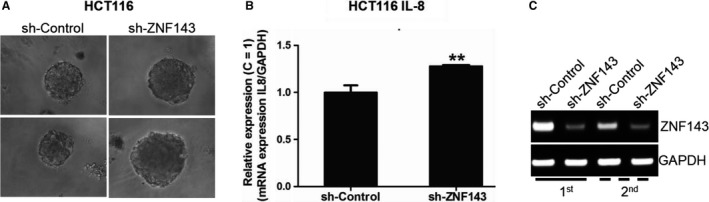
ZNF143 knockdown in colon cancer cells affects tumour cell growth in three‐dimensional (3D) culture system and IL‐8 expression. (A) HCT116 cells show better spheroid growth pattern in 3D culture using Matrigel. (B, C) Spheroids were harvested and analysed by reverse transcription (RT)‐polymerase chain reaction (PCR) to examine IL‐8 and ZNF143 expression. Results shown are representative of at least three independent experiments

We further investigated whether ZNF143 knockdown had an effect on IL‐8 expression in colon cancer cells. HCT116 and HT29 cells expressing sh‐ZNF143 were prepared as described previously.^20^ Cell lysates and supernatants were harvested to assess IL‐8 synthesis and secretion, respectively, by performing a CBA assay (Figure 1C,D). The results were normalized by protein quantification or cell number. Increased IL‐8 was observed in HCT116 and HT29 sh‐ZNF143 cells when compared to control cells. This result was confirmed by immunoblotting (Figure 1E) and real‐time PCR (Figure 1F) to assess the protein and RNA levels, respectively, of IL‐8. Furthermore, the expression of the human ZNF143 gene by transfecting a plasmid‐encoding full‐length human ZNF143 (pFLAG‐CMV‐hZNF143FL) reversed these effects according to RT‐PCR (Figure 1G), implying that the transcription of IL‐8 may be regulated by ZNF143 in colon cancer cells. To validate the effect of ZNF143 knockdown on IL‐8 expression and related signalling in colon cancer cells further, we established three‐dimensional (3D) spheroid culture system of tumour cells using Matrigel as described in Materials and Methods section (Figure 2A‐C). Regardless of ZNF143 expression level, cells have grown similarly in Matrigel at the beginning; however, spheroids from HCT116 sh‐ZNF143 cells were getting bigger in size and more in numbers than them from HCT116 sh‐Control cells, implying a role of ZNF143 for 3D growth (Figure 2A). Also, IL‐8 showed to be expressed more in colon cancer cells with less ZNF143, similar to data from monolayer culture. Regardless of growing system, ZNF143 expression showed to affect IL‐8 expression, suggesting a role of ZNF143 for cytokine regulation for tumour progression (Figure 2B,C).

### 3.2 ERK and JAK/STAT pathways are altered following ZNF143 knockdown and contribute to IL‐8 expression in colon cancer cells

**Figure 3:**
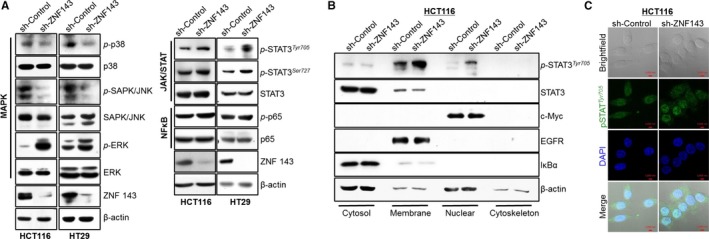
ZNF143 knockdown in colon cancer cells primarily affects extracellular signal‐regulated kinase (ERK)/ and JAK2/STAT3 signalling pathways. (A) Cells were harvested and analysed by immunoblotting to examine signalling pathway components. (B, C) Cells were harvested and subcellular fractionation (B) and immunostaining (C) were performed to determine STAT3 activation. Images were taken with the Zeiss 780 confocal microscope. Results shown are representative of at least three independent experiments

As the IL‐8 promoter contains consensus binding sites for nuclear factor kappa B (NF‐κB), β‐catenin/Tcf, hypoxia‐inducible factor (HIF)‐1 and activator protein (AP)‐1,^32^, ^33^, ^34^, several signalling pathways were investigated by immunoblotting (Figure 3A). Interestingly, the phosphorylation of ERKs and STAT3 was increased in HCT116 and HT29 sh‐ZNF143 cells when compared to control cells. In addition to the phosphorylation of Ser^727^ and/or Tyr^705^ of STAT3, nuclear translocation of activated STAT3 is required for transcriptional activation.^35^, ^36^, ^37^ Thus, translocation of activated *p*‐STAT3 into the nucleus was examined by the subcellular fractionation and immunofluorescence staining of colon cancer cells (Figure 3B,C). IκBα, epidermal growth factor receptor (EGFR) and c‐Myc served as controls for the cytosolic, membrane and nuclear fractions respectively. *p*‐STAT3^Tyr705^ was strongly detected in the membrane and nuclear fractions of HCT116 sh‐ZNF143 cells, and this was further confirmed by fluorescence staining in the nuclear region of HCT116 sh‐ZNF143 cells (Figure 3C), implying ZNF143 may play a role in STAT3 activation by enhancing the phosphorylation and translocation of STAT3. Phosphorylation of p38 kinase and c‐Jun amino terminal kinases (JNKs) were reduced in cells expressing sh‐ZNF143. Phosphorylation of RelA, the NF‐κB p65 subunit, was not significantly altered.

**Figure 4:**
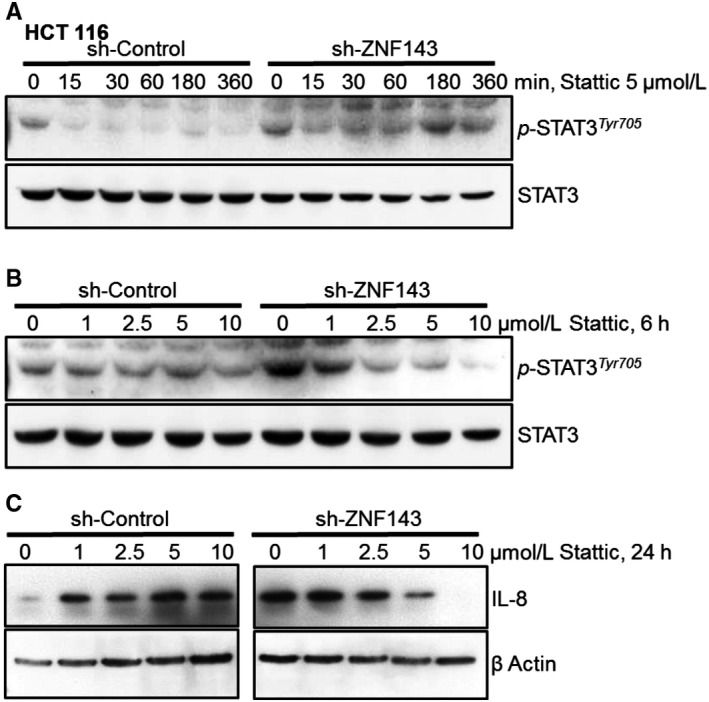
Stattic, a STAT3 inhibitor, attenuates IL‐8 expression in colon cancer cells in a ZNF143‐dependent manner. (A) Cells were treated with 5 μmol/L Stattic for the indicated time periods and harvested to determine STAT3 phosphorylation by immunoblotting. (B, C) Cells were treated with 0, 1, 2.5, 5, and 10 μmol/L Stattic for 6 hours (B) or 24 hours (C) and harvested to determine STAT3 phosphorylation (B) or IL‐8 expression (C) by immunoblotting. Results shown are representative of at least three independent experiments

To identify the effects of the ERK and JAK/STAT3 pathways on IL‐8 expression, HCT116 sh‐Control and sh‐ZNF143 cells were grown in the presence or absence of PD98059, which inhibits ERK activation and IL‐8 was measured in the media or protein extracts using the CBA assay (data not shown). Although treatment with PD98059 reduced the synthesis and secretion of IL‐8, the effect was not sufficient to identify a role for ERK in the regulation of IL‐8 expression in ZNF143‐knockdown cells. Stattic, a small molecule inhibitor, has been shown to selectively inhibit STAT3 through the SH2 domain regardless of the STAT3 activation state.^38^ We further confirmed the effect of STAT3 activation on IL‐8 expression in colon cancer cells with Stattic (Figure 4). Stattic disrupted the activation of STAT3 in a time‐ and dose‐dependent manner, resulting in decreased IL‐8 expression, suggesting a role of STAT3 for IL‐8 expression in colon cancer cell with less ZNF143.

### 3.3 IL‐8 may contribute to cell‐cell communication in the tumour microenvironment in colon cancer progression

**Figure 5:**
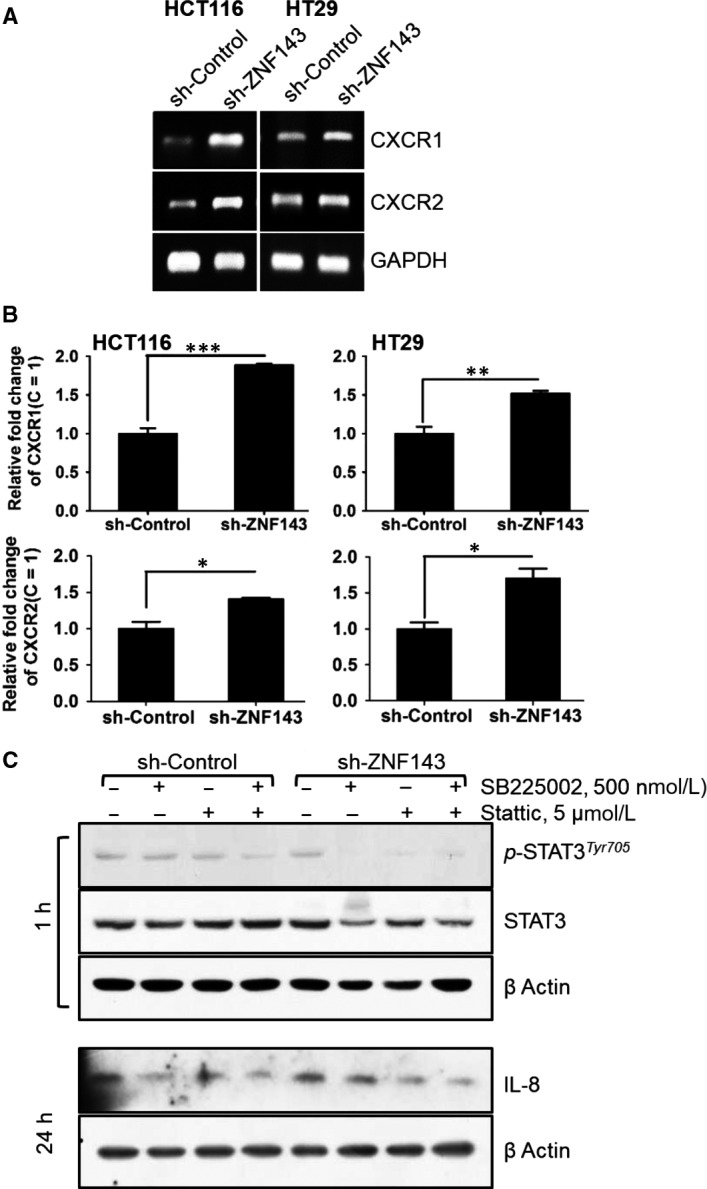
Increased IL‐8 contributes to STAT3 activation and IL‐8 expression through its receptor, CXCR2, in colon cancer cells. (A, B) Cells were harvested and analysed for mRNA expression of IL‐8 by reverse transcription (RT)‐polymerase chain reaction (PCR) (A) or real‐time PCR (B). (C) Cells were harvested, and the expression of signalling pathway components was determined by immunoblotting. Cells were treated with SB225002, an inhibitor of CXCR1, Stattic, or DMSO (vehicle) for 6 hours prior to harvesting. Results shown are representative of at least three independent experiments. Data are expressed as means ± S.E. of at least three independent experiments. Statistical significance was assessed using unpaired Student's *t* tests (***P < 0.0001 and *P < 0.002)

**Figure 6:**
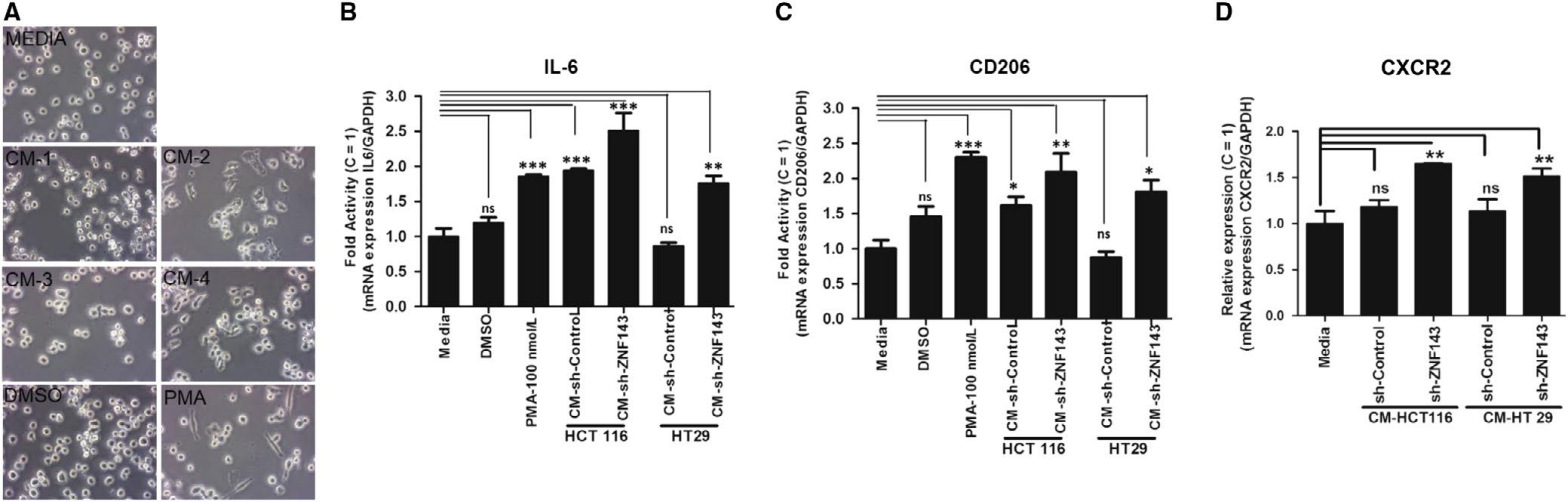
Increased IL‐8 in colon cancer cells with less ZNF143 communicate with immune cells for maintaining tumour microenvironment. (A‐D) THP‐1 cells were incubated with media or CM from cancer cells lines as shown in figure for 24 hours and observed for phenotypic changes during macrophage differentiation by light microscopy (A) and harvested for RNA isolation followed reverse transcription (RT)‐polymerase chain reaction (PCR) for expression of macrophage differentiation markers IL‐6 (B) and CD206 (C), IL‐8 receptor, CXCR2 (D). Some cells were treated with 100 nmol/L phorbol 12‐myristate13‐acetate (PMA) or DMSO for 24 hours. All data were analysed with the data from media as control, C. Results shown are representative of at least three independent experiments. Data are expressed as means ± S.E. of at least three independent experiments. Statistical significance was assessed using unpaired student's *t* test and one‐way ANOVA (***P < 0.0001; **P < 0.001 and *P < 0.01)

Within the tumour microenvironment, IL‐8 signalling is initiated by binding of its G‐protein‐coupled receptors CXCR1/2.^33^, ^39^ To examine whether ZNF143 expression affects on IL‐8 signalling in cancer cells, we examined the mRNA expression of CXCR1/2 (Figure 5A,B). HCT116 and HT29 cells expressed CXCR1/2, and this expression was increased following ZNF143 knockdown, implying that the expression of ZNF143 is important for the expression of CXCR1/2. SB225002, an antagonist of CXCR2, reduced Tyr^705^ phosphorylation of STAT3 and IL‐8 protein expression in HCT116 colon cancer cells, which was also apparent in HCT116 sh‐ZNF143 cells. To further investigate whether ZNF143 expression affects intercellular communication for pro‐tumoural microenvironments, we have grown THP‐1, human monocyte, in the presence of conditioned media (CM, combined regular growing media with media from colon cancer cells (1:1)) from cancer cells for 24 hours and observed the morphological alteration of THP‐1 cells (Figure 6A). THP‐1 cells treated with PMA^40^ showed morphological alteration, similarly THP‐1 cells incubated with CM from cells with less ZNF143, supporting the effect of ZNF143 knockdown on intercellular communication. CD206^41^ and IL‐6^42^ were shown to be expressed more in cells with CM‐cells with less ZNF143 than in cells with CM‐control cells (Figure 6B,C), implying factors from ZNF143 knockdown cells for pro‐tumoural tumour microenvironments. In accordance with Figure 5A,B, increased expression of IL‐8 receptor, CXCR2 was seen when THP‐1 cells was grown in CM‐cells with less ZNF143 than control cells (Figure 6D).

**Figure 7:**
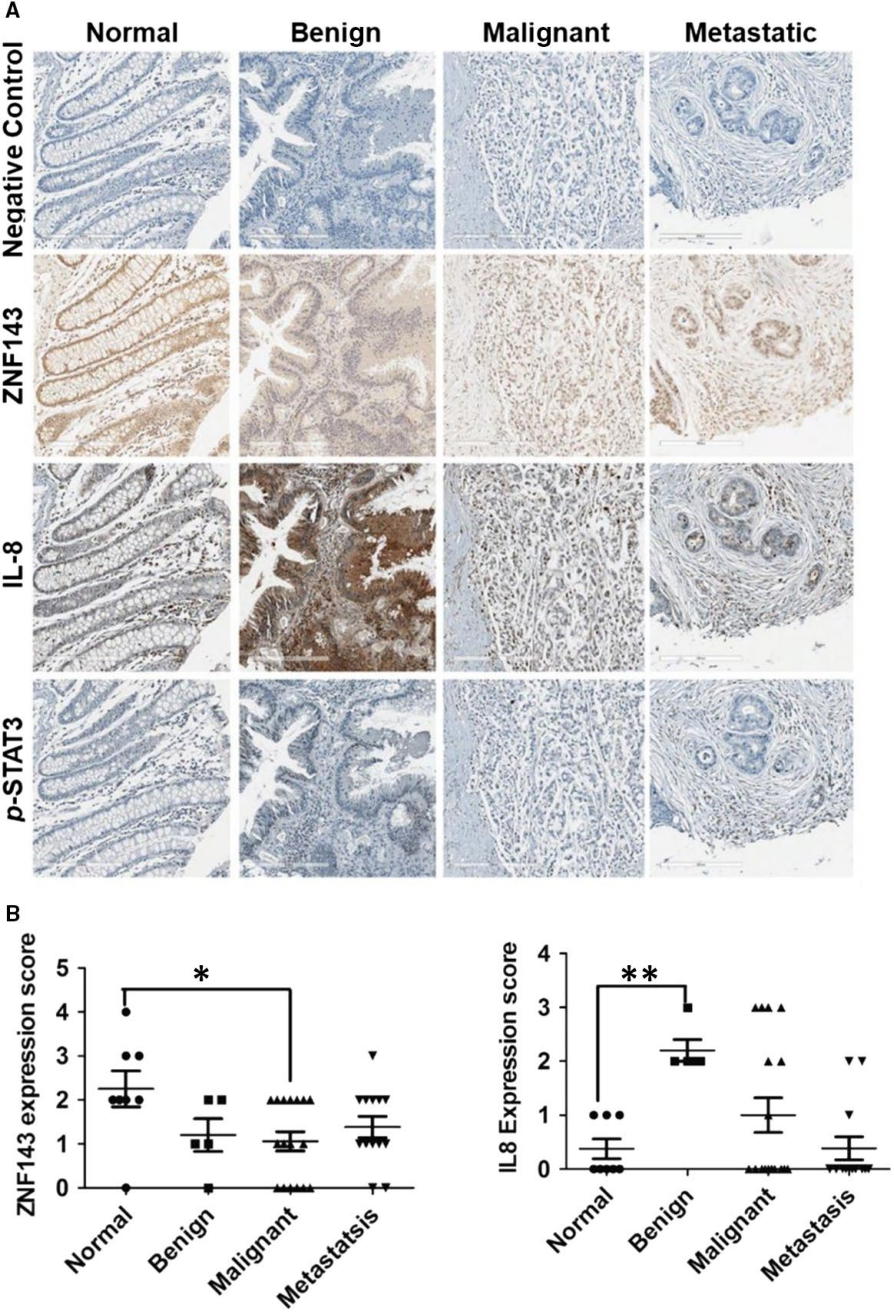
ZNF143‐IL‐8 cascade contributes to colon cancer progression. (A, B) Expression of ZNF143 and IL‐8 in colon cancer and normal tissue was examined by immunohistochemistry and scored by a professional pathologist. (A) The stained tissues on the tissue microarray were digitized at 20× magnification using the Aperio AT Turbo whole slide scanner equipped with a clinical grade RGB camera. (B) The results were categorized as normal, benign, malignant or metastatic according to information from the tissue microarray suppliers. Data are expressed as means ± S. E. of at least three independent experiments. Statistical significance was assessed using unpaired Student's *t* tests (*P < 0.006 and **P < 0.05)

Colon tissues on tissue arrays were stained with haematoxylin and specific antibodies against ZNF143, IL‐8, and *p*‐STAT3^Tyr705^ and scored by professional pathologists (Figure 7A,B, Tables S2 and S3). Stained tissues were digitized with the Aperio AT Turbo whole slide scanner. Nuclear expression of ZNF143 was reduced as tumour malignancy increased in colon cancer tissue. Interestingly, IL‐8 expression was higher in benign tissue when compared to normal tissue. In particular, some tissues showed reciprocal expression of ZNF143 and IL‐8 (Figure 7A), supporting a relationship between ZNF143 and IL‐8.

## 4 DISCUSSION

Increased cell motility has been observed in colon and breast cancer cells following a decrease in ZNF143 expression.^20^, ^24^ Another study demonstrated that overexpression of ZNF143 in gastric cancer cell lines enhanced metastatic potential.^43^ Strong expression of ZNF143 in lung adenocarcinomas was shown to predict shorter disease‐specific survival with increased Ki‐67 labelling.^21^ Gonzalez and colleagues showed that ZNF143 as an important regulator for CCAAT/enhancer‐binding protein alpha (C/EBPα) in myeloid cells,^23^ whose dysregulation is closely regulated to myeloid leukaemia. Although many controversies surround the relationship between the expression of ZNF143 and cancer malignancy, all the evidence to date suggests that ZNF143 plays a role in cancer development in various experimental systems. In this study, we have been suggested that ZNF143 expression may be important in the tumour microenvironment by regulating secreted molecules, such as cytokines, which may communicate with other cells. Our findings demonstrate that reduced expression of ZNF143 enhanced the expression and secretion of IL‐8 by modulating the ERK/STAT3/IL‐8 signalling axis. Our data suggest a ZNF143‐dependent signalling mechanism that can modulate the autocrine activity of pro‐inflammatory cytokines to promote colon cancer progression.

IL‐8 is widely produced by human cancer cells in numerous malignant diseases and exhibits both paracrine and autocrine effects. The paracrine signalling modulates the migration of endothelial cells and promotes the function of IL‐8 as a chemoattractant for neutrophils, whereas the autocrine signalling promotes the growth of different cancer cell types.^44^ IL‐8, a key factor produced by cancer cells, is important for maintenance of the tumour microenvironment, myeloid‐derived suppressor cell recruitment, myofibroblast expansion and tumour angiogenesis.^45^ Recently, IL‐8 has been shown to increase the mobilization of immature myeloid cells, resulting in increased tumourigenesis.^46^ In this study, we investigated the key factors altered following knockdown of ZNF143 by proteomic profiling in HCT116 cell supernatants. Among cytokines, IL‐8 attracted attention, with its significantly increased levels, as a secreted soluble factor in ZNF143‐knockdown colon cancer cells (Figure 1). In addition to IL‐8, we also found that expression of the chemokines MIF and IL‐10 (Figure 1A,B), which have been shown to mediate tumour‐promoting crosstalk between tumours and the tumour microenvironment, was increased following ZNF143 knockdown. IL‐8 signalling has been linked to the MAPK signalling cascade, which leads to the downstream phosphorylation of ERK1/2 (Figure 3A). Recently, Abdul Aziz et al reported that MIF, via interaction with its receptor, increases IL‐8 expression in the tumour microenvironment,^47^ which recruits non‐receptor tyrosine kinases for phosphorylation of ERK1/2.^48^ IL‐10 inhibits production of TNF‐α and thus inhibits NF‐κB while activating JAK/STAT3 signalling (Figure 3A‐C) by binding the IL‐10 receptor α and β chain.^49^ Therefore, it is likely that dysregulation of ZNF143 also promotes formation of the tumourigenic microenvironment in a paracrine manner to modulate the initiation and progression of colon cancer.

IL‐8 signalling is mediated through the G‐protein coupled receptors CXCR1/2 and has been associated with a poor prognosis in colon cancer.^50^, ^51^ It can also activate STAT3, AKT, ERK and EGFR signalling, which can enhance migration and inhibit anoikis of tumour cells.^52^ Our results demonstrated the involvement in STAT3 signalling (Figure 3) with increased expression of CXCR1/2 (Figure 5A,B), resulting in the overexpression of IL‐8 in ZNF143‐silenced cells (Figure 5C). The IL‐8 promoter containing binding sites for NF‐κB and TNFα is a well‐known inducer of NF‐κB.^53^, ^54^ We also observed that TNFα significantly induced the phosphorylation of p65 in ZNF143‐silenced cells (data not shown). Subsequent inhibition of STAT3 signalling reduced TNFα‐induced activation of p65, suggesting a connection between the STAT3 and NF‐κB pathways.

Interestingly, inhibition of STAT3 signalling resulted in reduced IL‐8 signalling, whereas STAT3 phosphorylation was decreased when IL‐8 signalling was inhibited with SB225002. On the other hand, treatment of ZNF143‐silenced cells with both inhibitors potentially inhibited the expression of either IL‐8 or STAT3 compared with the control, indicating ZNF143 may regulate the STAT3/IL‐8/CXCR1/2 signalling axis (Figure 5C). Finally, we identified a negative correlation between ZNF143 and IL‐8 in the tissues of colon cancer patients, suggesting it may be of diagnostic importance (Figure 7).

Tumour‐associated macrophages are one of the widely found host immune cells in the tumour microenvironment involved in different cellular events to facilitate metastasis.^6^ In our study, we found ZNF143 reduced colon cancer cells secrete some factors including IL‐8, potentially differentiated monocytes into macrophages with increased expression of macrophage markers IL‐6 and CD206 (Figure 6), suggesting its role in intercellular communication with immune cells to set up an environment for tumour progression to metastasis.

Hence, this is the first study focused on ZNF143 and the regulation of IL‐8, which may be important for maintaining the tumour microenvironment and facilitating metastasis in colon cancer cells.

## CONFLICT OF INTEREST

The authors have no conflict of interests.

## AUTHORS’ CONTRIBUTIONS

VV, AP and HY performed the research; HY designed the research study; VV, AP and BC contributed essential reagents or tools; VV and EH analysed the data; VV and HY wrote the manuscript.
