# Supplementary material for: Loss of zinc‐finger protein 143 contributes to tumour progression by interleukin‐8‐CXCR axis in colon cancer
Source: J Cell Mol Med. 2019 Apr 1;23(6):4043–53. doi: 10.1111/jcmm.14290 (PMC6533486; doi:10.1111/jcmm.14290)
Supplement: Supplementary file 1 [file JCMM-23-4043-s001.pptx]

## Slide 1
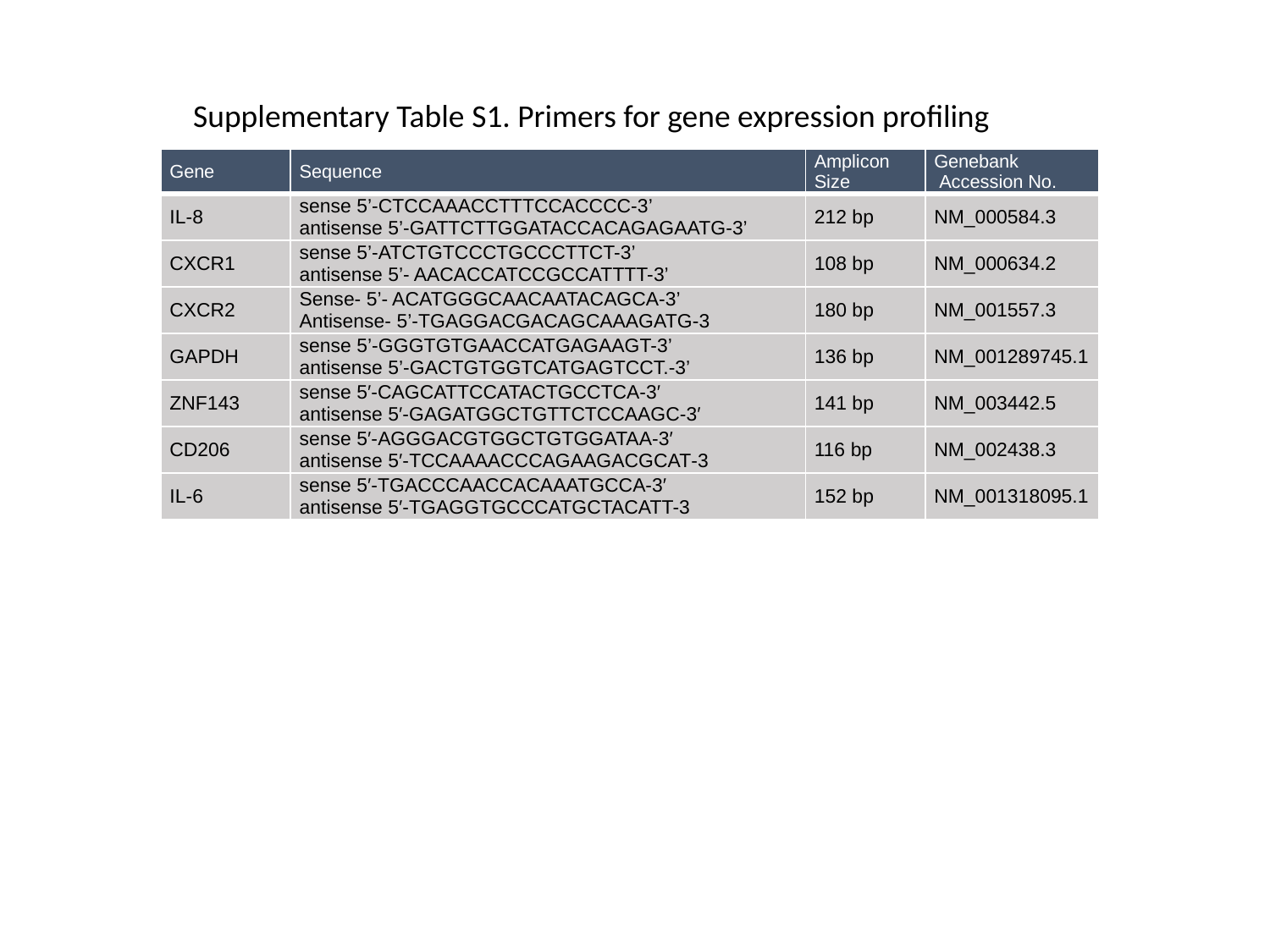

Supplementary Table S1. Primers for gene expression profiling
| Gene | Sequence | Amplicon Size | Genebank Accession No. |
| --- | --- | --- | --- |
| IL-8 | sense 5’-CTCCAAACCTTTCCACCCC-3’ antisense 5’-GATTCTTGGATACCACAGAGAATG-3’ | 212 bp | NM\_000584.3 |
| CXCR1 | sense 5’-ATCTGTCCCTGCCCTTCT-3’ antisense 5’- AACACCATCCGCCATTTT-3’ | 108 bp | NM\_000634.2 |
| CXCR2 | Sense- 5’- ACATGGGCAACAATACAGCA-3’ Antisense- 5’-TGAGGACGACAGCAAAGATG-3 | 180 bp | NM\_001557.3 |
| GAPDH | sense 5’-GGGTGTGAACCATGAGAAGT-3’ antisense 5’-GACTGTGGTCATGAGTCCT.-3’ | 136 bp | NM\_001289745.1 |
| ZNF143 | sense 5′-CAGCATTCCATACTGCCTCA-3′ antisense 5′-GAGATGGCTGTTCTCCAAGC-3′ | 141 bp | NM\_003442.5 |
| CD206 | sense 5′-AGGGACGTGGCTGTGGATAA-3′ antisense 5′-TCCAAAACCCAGAAGACGCAT-3 | 116 bp | NM\_002438.3 |
| IL-6 | sense 5′-TGACCCAACCACAAATGCCA-3′ antisense 5′-TGAGGTGCCCATGCTACATT-3 | 152 bp | NM\_001318095.1 |

## Slide 2
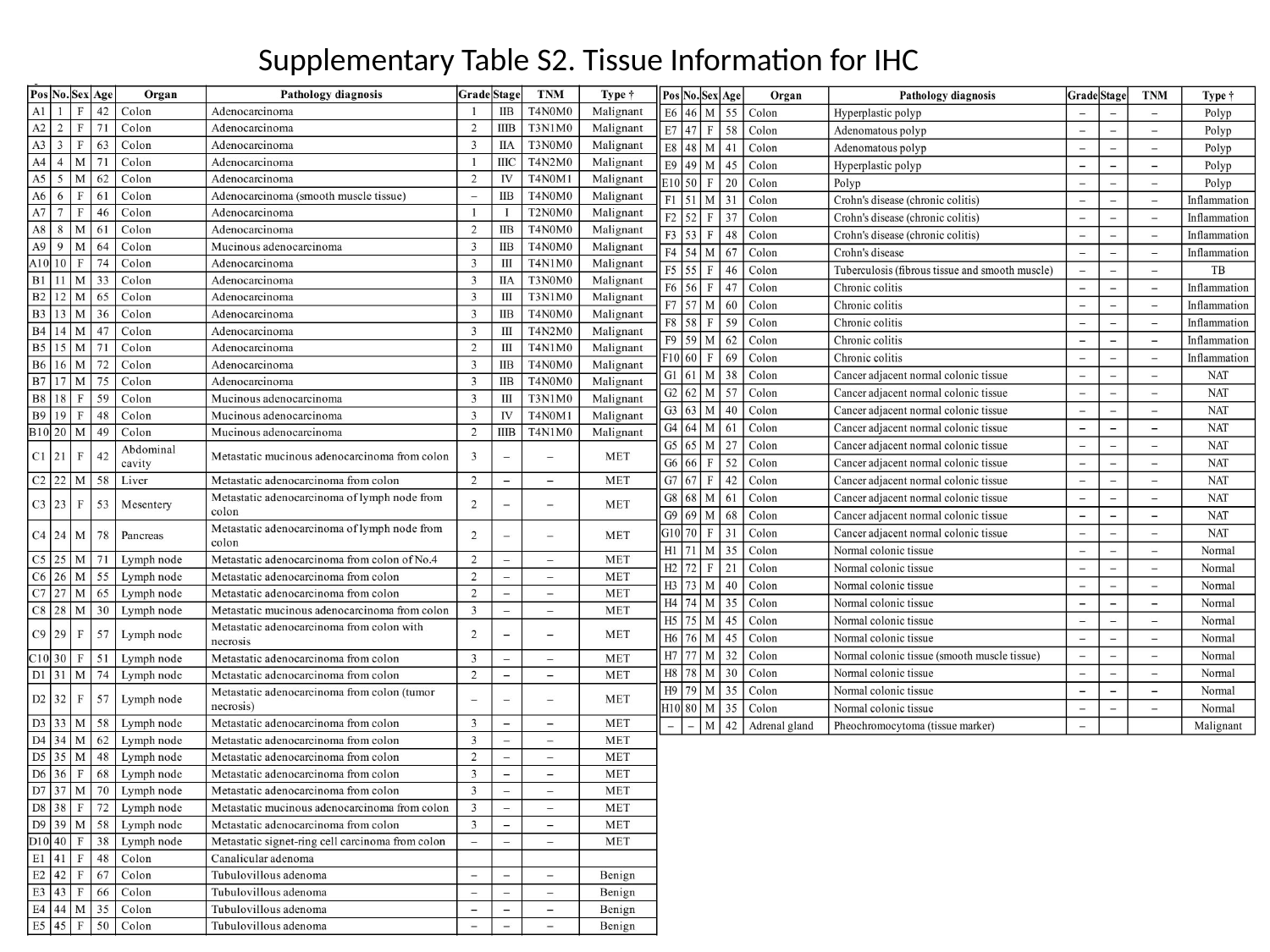

Supplementary Table S2. Tissue Information for IHC

## Slide 3
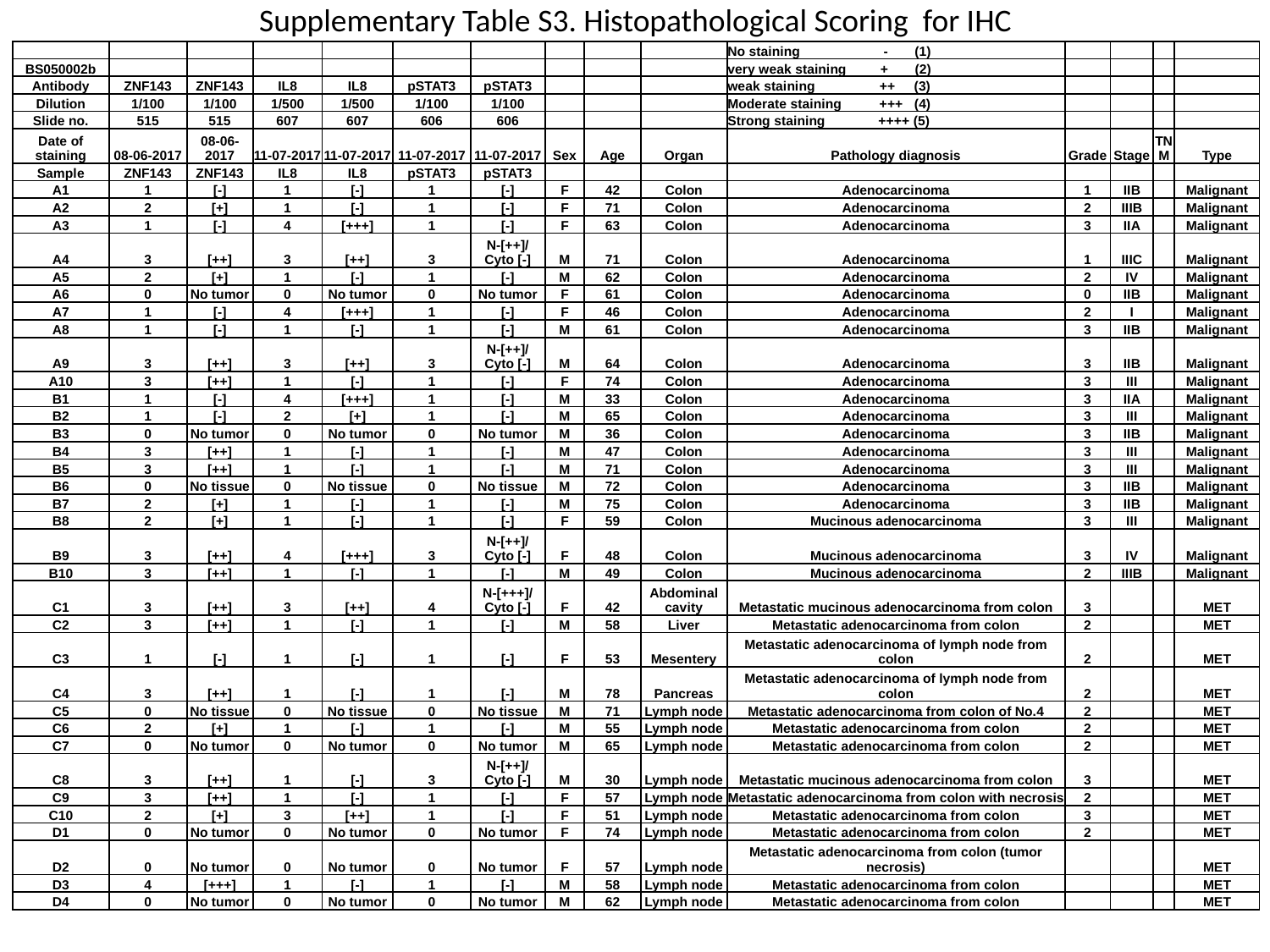

Supplementary Table S3. Histopathological Scoring for IHC
| | | | | | | | | | | No staining - (1) | | | | |
| --- | --- | --- | --- | --- | --- | --- | --- | --- | --- | --- | --- | --- | --- | --- |
| BS050002b | | | | | | | | | | very weak staining + (2) | | | | |
| Antibody | ZNF143 | ZNF143 | IL8 | IL8 | pSTAT3 | pSTAT3 | | | | weak staining ++ (3) | | | | |
| Dilution | 1/100 | 1/100 | 1/500 | 1/500 | 1/100 | 1/100 | | | | Moderate staining +++ (4) | | | | |
| Slide no. | 515 | 515 | 607 | 607 | 606 | 606 | | | | Strong staining ++++ (5) | | | | |
| Date of staining | 08-06-2017 | 08-06-2017 | 11-07-2017 | 11-07-2017 | 11-07-2017 | 11-07-2017 | Sex | Age | Organ | Pathology diagnosis | Grade | Stage | TNM | Type |
| Sample | ZNF143 | ZNF143 | IL8 | IL8 | pSTAT3 | pSTAT3 | | | | | | | | |
| A1 | 1 | [-] | 1 | [-] | 1 | [-] | F | 42 | Colon | Adenocarcinoma | 1 | IIB | | Malignant |
| A2 | 2 | [+] | 1 | [-] | 1 | [-] | F | 71 | Colon | Adenocarcinoma | 2 | IIIB | | Malignant |
| A3 | 1 | [-] | 4 | [+++] | 1 | [-] | F | 63 | Colon | Adenocarcinoma | 3 | IIA | | Malignant |
| A4 | 3 | [++] | 3 | [++] | 3 | N-[++]/ Cyto [-] | M | 71 | Colon | Adenocarcinoma | 1 | IIIC | | Malignant |
| A5 | 2 | [+] | 1 | [-] | 1 | [-] | M | 62 | Colon | Adenocarcinoma | 2 | IV | | Malignant |
| A6 | 0 | No tumor | 0 | No tumor | 0 | No tumor | F | 61 | Colon | Adenocarcinoma | 0 | IIB | | Malignant |
| A7 | 1 | [-] | 4 | [+++] | 1 | [-] | F | 46 | Colon | Adenocarcinoma | 2 | I | | Malignant |
| A8 | 1 | [-] | 1 | [-] | 1 | [-] | M | 61 | Colon | Adenocarcinoma | 3 | IIB | | Malignant |
| A9 | 3 | [++] | 3 | [++] | 3 | N-[++]/ Cyto [-] | M | 64 | Colon | Adenocarcinoma | 3 | IIB | | Malignant |
| A10 | 3 | [++] | 1 | [-] | 1 | [-] | F | 74 | Colon | Adenocarcinoma | 3 | III | | Malignant |
| B1 | 1 | [-] | 4 | [+++] | 1 | [-] | M | 33 | Colon | Adenocarcinoma | 3 | IIA | | Malignant |
| B2 | 1 | [-] | 2 | [+] | 1 | [-] | M | 65 | Colon | Adenocarcinoma | 3 | III | | Malignant |
| B3 | 0 | No tumor | 0 | No tumor | 0 | No tumor | M | 36 | Colon | Adenocarcinoma | 3 | IIB | | Malignant |
| B4 | 3 | [++] | 1 | [-] | 1 | [-] | M | 47 | Colon | Adenocarcinoma | 3 | III | | Malignant |
| B5 | 3 | [++] | 1 | [-] | 1 | [-] | M | 71 | Colon | Adenocarcinoma | 3 | III | | Malignant |
| B6 | 0 | No tissue | 0 | No tissue | 0 | No tissue | M | 72 | Colon | Adenocarcinoma | 3 | IIB | | Malignant |
| B7 | 2 | [+] | 1 | [-] | 1 | [-] | M | 75 | Colon | Adenocarcinoma | 3 | IIB | | Malignant |
| B8 | 2 | [+] | 1 | [-] | 1 | [-] | F | 59 | Colon | Mucinous adenocarcinoma | 3 | III | | Malignant |
| B9 | 3 | [++] | 4 | [+++] | 3 | N-[++]/ Cyto [-] | F | 48 | Colon | Mucinous adenocarcinoma | 3 | IV | | Malignant |
| B10 | 3 | [++] | 1 | [-] | 1 | [-] | M | 49 | Colon | Mucinous adenocarcinoma | 2 | IIIB | | Malignant |
| C1 | 3 | [++] | 3 | [++] | 4 | N-[+++]/ Cyto [-] | F | 42 | Abdominal cavity | Metastatic mucinous adenocarcinoma from colon | 3 | | | MET |
| C2 | 3 | [++] | 1 | [-] | 1 | [-] | M | 58 | Liver | Metastatic adenocarcinoma from colon | 2 | | | MET |
| C3 | 1 | [-] | 1 | [-] | 1 | [-] | F | 53 | Mesentery | Metastatic adenocarcinoma of lymph node from colon | 2 | | | MET |
| C4 | 3 | [++] | 1 | [-] | 1 | [-] | M | 78 | Pancreas | Metastatic adenocarcinoma of lymph node from colon | 2 | | | MET |
| C5 | 0 | No tissue | 0 | No tissue | 0 | No tissue | M | 71 | Lymph node | Metastatic adenocarcinoma from colon of No.4 | 2 | | | MET |
| C6 | 2 | [+] | 1 | [-] | 1 | [-] | M | 55 | Lymph node | Metastatic adenocarcinoma from colon | 2 | | | MET |
| C7 | 0 | No tumor | 0 | No tumor | 0 | No tumor | M | 65 | Lymph node | Metastatic adenocarcinoma from colon | 2 | | | MET |
| C8 | 3 | [++] | 1 | [-] | 3 | N-[++]/ Cyto [-] | M | 30 | Lymph node | Metastatic mucinous adenocarcinoma from colon | 3 | | | MET |
| C9 | 3 | [++] | 1 | [-] | 1 | [-] | F | 57 | Lymph node | Metastatic adenocarcinoma from colon with necrosis | 2 | | | MET |
| C10 | 2 | [+] | 3 | [++] | 1 | [-] | F | 51 | Lymph node | Metastatic adenocarcinoma from colon | 3 | | | MET |
| D1 | 0 | No tumor | 0 | No tumor | 0 | No tumor | F | 74 | Lymph node | Metastatic adenocarcinoma from colon | 2 | | | MET |
| D2 | 0 | No tumor | 0 | No tumor | 0 | No tumor | F | 57 | Lymph node | Metastatic adenocarcinoma from colon (tumor necrosis) | | | | MET |
| D3 | 4 | [+++] | 1 | [-] | 1 | [-] | M | 58 | Lymph node | Metastatic adenocarcinoma from colon | | | | MET |
| D4 | 0 | No tumor | 0 | No tumor | 0 | No tumor | M | 62 | Lymph node | Metastatic adenocarcinoma from colon | | | | MET |

## Slide 4
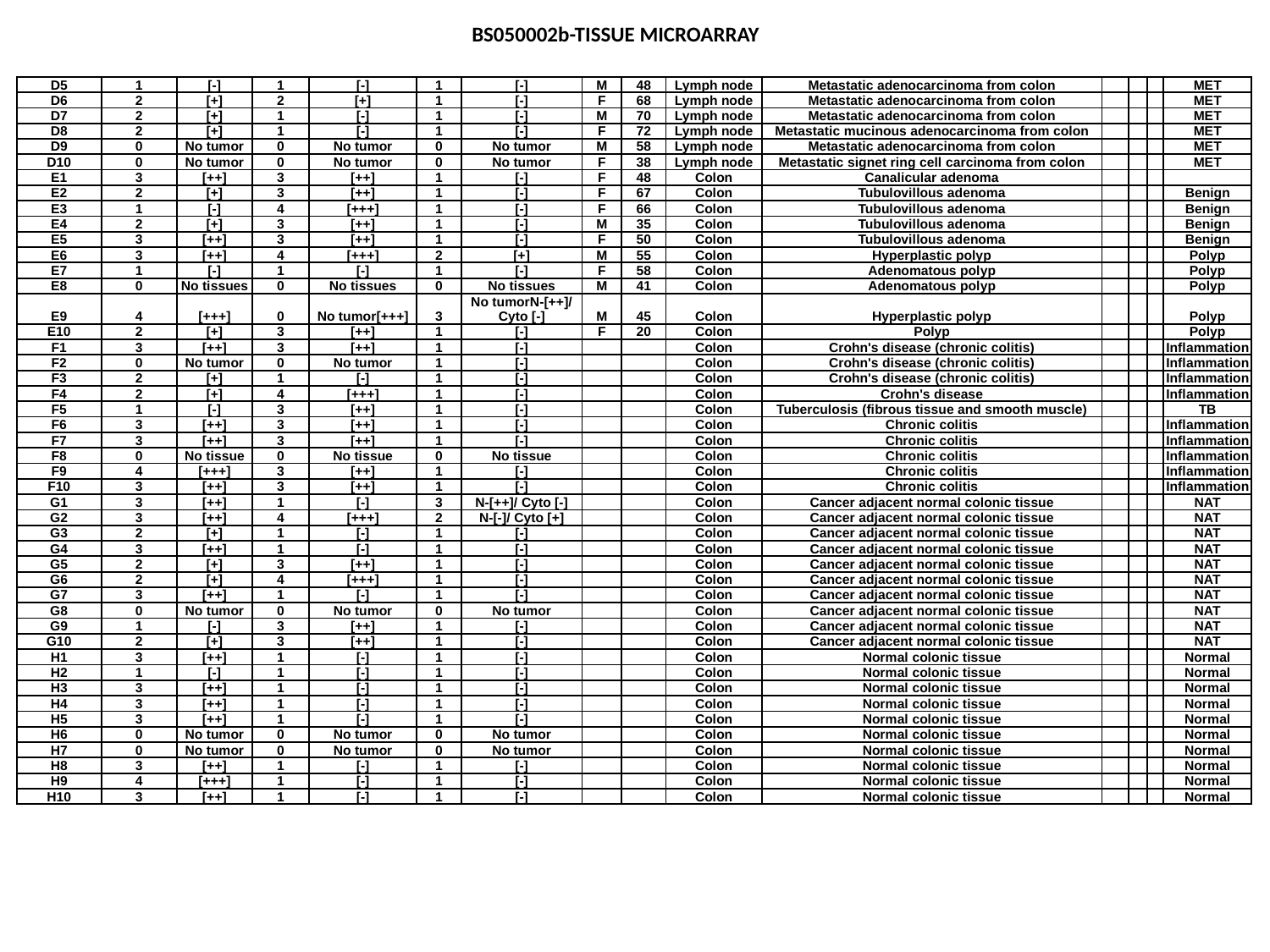

BS050002b-TISSUE MICROARRAY
| D5 | 1 | [-] | 1 | [-] | 1 | [-] | M | 48 | Lymph node | Metastatic adenocarcinoma from colon | | | | MET |
| --- | --- | --- | --- | --- | --- | --- | --- | --- | --- | --- | --- | --- | --- | --- |
| D6 | 2 | [+] | 2 | [+] | 1 | [-] | F | 68 | Lymph node | Metastatic adenocarcinoma from colon | | | | MET |
| D7 | 2 | [+] | 1 | [-] | 1 | [-] | M | 70 | Lymph node | Metastatic adenocarcinoma from colon | | | | MET |
| D8 | 2 | [+] | 1 | [-] | 1 | [-] | F | 72 | Lymph node | Metastatic mucinous adenocarcinoma from colon | | | | MET |
| D9 | 0 | No tumor | 0 | No tumor | 0 | No tumor | M | 58 | Lymph node | Metastatic adenocarcinoma from colon | | | | MET |
| D10 | 0 | No tumor | 0 | No tumor | 0 | No tumor | F | 38 | Lymph node | Metastatic signet ring cell carcinoma from colon | | | | MET |
| E1 | 3 | [++] | 3 | [++] | 1 | [-] | F | 48 | Colon | Canalicular adenoma | | | | |
| E2 | 2 | [+] | 3 | [++] | 1 | [-] | F | 67 | Colon | Tubulovillous adenoma | | | | Benign |
| E3 | 1 | [-] | 4 | [+++] | 1 | [-] | F | 66 | Colon | Tubulovillous adenoma | | | | Benign |
| E4 | 2 | [+] | 3 | [++] | 1 | [-] | M | 35 | Colon | Tubulovillous adenoma | | | | Benign |
| E5 | 3 | [++] | 3 | [++] | 1 | [-] | F | 50 | Colon | Tubulovillous adenoma | | | | Benign |
| E6 | 3 | [++] | 4 | [+++] | 2 | [+] | M | 55 | Colon | Hyperplastic polyp | | | | Polyp |
| E7 | 1 | [-] | 1 | [-] | 1 | [-] | F | 58 | Colon | Adenomatous polyp | | | | Polyp |
| E8 | 0 | No tissues | 0 | No tissues | 0 | No tissues | M | 41 | Colon | Adenomatous polyp | | | | Polyp |
| E9 | 4 | [+++] | 0 | No tumor[+++] | 3 | No tumorN-[++]/ Cyto [-] | M | 45 | Colon | Hyperplastic polyp | | | | Polyp |
| E10 | 2 | [+] | 3 | [++] | 1 | [-] | F | 20 | Colon | Polyp | | | | Polyp |
| F1 | 3 | [++] | 3 | [++] | 1 | [-] | | | Colon | Crohn's disease (chronic colitis) | | | | Inflammation |
| F2 | 0 | No tumor | 0 | No tumor | 1 | [-] | | | Colon | Crohn's disease (chronic colitis) | | | | Inflammation |
| F3 | 2 | [+] | 1 | [-] | 1 | [-] | | | Colon | Crohn's disease (chronic colitis) | | | | Inflammation |
| F4 | 2 | [+] | 4 | [+++] | 1 | [-] | | | Colon | Crohn's disease | | | | Inflammation |
| F5 | 1 | [-] | 3 | [++] | 1 | [-] | | | Colon | Tuberculosis (fibrous tissue and smooth muscle) | | | | TB |
| F6 | 3 | [++] | 3 | [++] | 1 | [-] | | | Colon | Chronic colitis | | | | Inflammation |
| F7 | 3 | [++] | 3 | [++] | 1 | [-] | | | Colon | Chronic colitis | | | | Inflammation |
| F8 | 0 | No tissue | 0 | No tissue | 0 | No tissue | | | Colon | Chronic colitis | | | | Inflammation |
| F9 | 4 | [+++] | 3 | [++] | 1 | [-] | | | Colon | Chronic colitis | | | | Inflammation |
| F10 | 3 | [++] | 3 | [++] | 1 | [-] | | | Colon | Chronic colitis | | | | Inflammation |
| G1 | 3 | [++] | 1 | [-] | 3 | N-[++]/ Cyto [-] | | | Colon | Cancer adjacent normal colonic tissue | | | | NAT |
| G2 | 3 | [++] | 4 | [+++] | 2 | N-[-]/ Cyto [+] | | | Colon | Cancer adjacent normal colonic tissue | | | | NAT |
| G3 | 2 | [+] | 1 | [-] | 1 | [-] | | | Colon | Cancer adjacent normal colonic tissue | | | | NAT |
| G4 | 3 | [++] | 1 | [-] | 1 | [-] | | | Colon | Cancer adjacent normal colonic tissue | | | | NAT |
| G5 | 2 | [+] | 3 | [++] | 1 | [-] | | | Colon | Cancer adjacent normal colonic tissue | | | | NAT |
| G6 | 2 | [+] | 4 | [+++] | 1 | [-] | | | Colon | Cancer adjacent normal colonic tissue | | | | NAT |
| G7 | 3 | [++] | 1 | [-] | 1 | [-] | | | Colon | Cancer adjacent normal colonic tissue | | | | NAT |
| G8 | 0 | No tumor | 0 | No tumor | 0 | No tumor | | | Colon | Cancer adjacent normal colonic tissue | | | | NAT |
| G9 | 1 | [-] | 3 | [++] | 1 | [-] | | | Colon | Cancer adjacent normal colonic tissue | | | | NAT |
| G10 | 2 | [+] | 3 | [++] | 1 | [-] | | | Colon | Cancer adjacent normal colonic tissue | | | | NAT |
| H1 | 3 | [++] | 1 | [-] | 1 | [-] | | | Colon | Normal colonic tissue | | | | Normal |
| H2 | 1 | [-] | 1 | [-] | 1 | [-] | | | Colon | Normal colonic tissue | | | | Normal |
| H3 | 3 | [++] | 1 | [-] | 1 | [-] | | | Colon | Normal colonic tissue | | | | Normal |
| H4 | 3 | [++] | 1 | [-] | 1 | [-] | | | Colon | Normal colonic tissue | | | | Normal |
| H5 | 3 | [++] | 1 | [-] | 1 | [-] | | | Colon | Normal colonic tissue | | | | Normal |
| H6 | 0 | No tumor | 0 | No tumor | 0 | No tumor | | | Colon | Normal colonic tissue | | | | Normal |
| H7 | 0 | No tumor | 0 | No tumor | 0 | No tumor | | | Colon | Normal colonic tissue | | | | Normal |
| H8 | 3 | [++] | 1 | [-] | 1 | [-] | | | Colon | Normal colonic tissue | | | | Normal |
| H9 | 4 | [+++] | 1 | [-] | 1 | [-] | | | Colon | Normal colonic tissue | | | | Normal |
| H10 | 3 | [++] | 1 | [-] | 1 | [-] | | | Colon | Normal colonic tissue | | | | Normal |
