# Supplementary material for: Loss of zinc‐finger protein 143 contributes to tumour progression by interleukin‐8‐CXCR axis in colon cancer
Source: J Cell Mol Med. 2019 Apr 1;23(6):4043–53. doi: 10.1111/jcmm.14290 (PMC6533486; doi:10.1111/jcmm.14290)
Supplement: Supplementary file 2 [file JCMM-23-4043-s002.pdf]

2018-09-21 18:20:27 11375

Supplementary Table S2. Tissue Information for IHC

Pos	No.	Sex	Age	Organ	Pathology diagnosis	Grade	Stage	TNM	Type †
A1	1	F	42	Colon	Adenocarcinoma	1	IIB	T4N0M0	Malignant
A2	2	F	71	Colon	Adenocarcinoma	2	IIIB	T3N1M0	Malignant
A3	3	F	63	Colon	Adenocarcinoma	3	IIA	T3N0M0	Malignant
A4	4	M	71	Colon	Adenocarcinoma	1	IIIC	T4N2M0	Malignant
A5	5	M	62	Colon	Adenocarcinoma	2	IV	T4N0M1	Malignant
A6	6	F	61	Colon	Adenocarcinoma (smooth muscle tissue)	–	IIB	T4N0M0	Malignant
A7	7	F	46	Colon	Adenocarcinoma	1	I	T2N0M0	Malignant
A8	8	M	61	Colon	Adenocarcinoma	2	IIB	T4N0M0	Malignant
A9	9	M	64	Colon	Mucinous adenocarcinoma	3	IIB	T4N0M0	Malignant
A10	10	F	74	Colon	Adenocarcinoma	3	III	T4N1M0	Malignant
B1	11	M	33	Colon	Adenocarcinoma	3	IIA	T3N0M0	Malignant
B2	12	M	65	Colon	Adenocarcinoma	3	III	T3N1M0	Malignant
B3	13	M	36	Colon	Adenocarcinoma	3	IIB	T4N0M0	Malignant
B4	14	M	47	Colon	Adenocarcinoma	3	III	T4N2M0	Malignant
B5	15	M	71	Colon	Adenocarcinoma	2	III	T4N1M0	Malignant
B6	16	M	72	Colon	Adenocarcinoma	3	IIB	T4N0M0	Malignant
B7	17	M	75	Colon	Adenocarcinoma	3	IIB	T4N0M0	Malignant
B8	18	F	59	Colon	Mucinous adenocarcinoma	3	III	T3N1M0	Malignant
B9	19	F	48	Colon	Mucinous adenocarcinoma	3	IV	T4N0M1	Malignant
B10	20	M	49	Colon	Mucinous adenocarcinoma	2	IIIB	T4N1M0	Malignant
C1	21	F	42	Abdominal cavity	Metastatic mucinous adenocarcinoma from colon	3	–	–	MET
C2	22	M	58	Liver	Metastatic adenocarcinoma from colon	2	–	–	MET
C3	23	F	53	Mesentery	Metastatic adenocarcinoma of lymph node from colon	2	–	–	MET
C4	24	M	78	Pancreas	Metastatic adenocarcinoma of lymph node from colon	2	–	–	MET
C5	25	M	71	Lymph node	Metastatic adenocarcinoma from colon of No.4	2	–	–	MET
C6	26	M	55	Lymph node	Metastatic adenocarcinoma from colon	2	–	–	MET
C7	27	M	65	Lymph node	Metastatic adenocarcinoma from colon	2	–	–	MET
C8	28	M	30	Lymph node	Metastatic mucinous adenocarcinoma from colon	3	–	–	MET
C9	29	F	57	Lymph node	Metastatic adenocarcinoma from colon with necrosis	2	–	–	MET
C10	30	F	51	Lymph node	Metastatic adenocarcinoma from colon	3	–	–	MET
D1	31	M	74	Lymph node	Metastatic adenocarcinoma from colon	2	–	–	MET
D2	32	F	57	Lymph node	Metastatic adenocarcinoma from colon (tumor necrosis)	–	–	–	MET
D3	33	M	58	Lymph node	Metastatic adenocarcinoma from colon	3	–	–	MET
D4	34	M	62	Lymph node	Metastatic adenocarcinoma from colon	3	–	–	MET
D5	35	M	48	Lymph node	Metastatic adenocarcinoma from colon	2	–	–	MET
D6	36	F	68	Lymph node	Metastatic adenocarcinoma from colon	3	–	–	MET
D7	37	M	70	Lymph node	Metastatic adenocarcinoma from colon	3	–	–	MET
D8	38	F	72	Lymph node	Metastatic mucinous adenocarcinoma from colon	3	–	–	MET
D9	39	M	58	Lymph node	Metastatic adenocarcinoma from colon	3	–	–	MET
D10	40	F	38	Lymph node	Metastatic signet-ring cell carcinoma from colon	–	–	–	MET
E1	41	F	48	Colon	Canalicular adenoma				
E2	42	F	67	Colon	Tubulovillous adenoma	–	–	–	Benign
E3	43	F	66	Colon	Tubulovillous adenoma	–	–	–	Benign
E4	44	M	35	Colon	Tubulovillous adenoma	–	–	–	Benign
E5	45	F	50	Colon	Tubulovillous adenoma	–	–	–	Benign
E6	46	M	55	Colon	Hyperplastic polyp	–	–	–	Polyp
E7	47	F	58	Colon	Adenomatous polyp	–	–	–	Polyp
E8	48	M	41	Colon	Adenomatous polyp	–	–	–	Polyp
E9	49	M	45	Colon	Hyperplastic polyp	–	–	–	Polyp
E10	50	F	20	Colon	Polyp	–	–	–	Polyp
F1	51	M	31	Colon	Crohn's disease (chronic colitis)	–	–	–	Inflammation
F2	52	F	37	Colon	Crohn's disease (chronic colitis)	–	–	–	Inflammation
F3	53	F	48	Colon	Crohn's disease (chronic colitis)	–	–	–	Inflammation
F4	54	M	67	Colon	Crohn's disease	–	–	–	Inflammation
F5	55	F	46	Colon	Tuberculosis (fibrous tissue and smooth muscle)	–	–	–	TB
F6	56	F	47	Colon	Chronic colitis	–	–	–	Inflammation
F7	57	M	60	Colon	Chronic colitis	–	–	–	Inflammation
F8	58	F	59	Colon	Chronic colitis	–	–	–	Inflammation
F9	59	M	62	Colon	Chronic colitis	–	–	–	Inflammation
F10	60	F	69	Colon	Chronic colitis	–	–	–	Inflammation
G1	61	M	38	Colon	Cancer adjacent normal colonic tissue	–	–	–	NAT
G2	62	M	57	Colon	Cancer adjacent normal colonic tissue	–	–	–	NAT
G3	63	M	40	Colon	Cancer adjacent normal colonic tissue	–	–	–	NAT
G4	64	M	61	Colon	Cancer adjacent normal colonic tissue	–	–	–	NAT
G5	65	M	27	Colon	Cancer adjacent normal colonic tissue	–	–	–	NAT
G6	66	F	52	Colon	Cancer adjacent normal colonic tissue	–	–	–	NAT
G7	67	F	42	Colon	Cancer adjacent normal colonic tissue	–	–	–	NAT
G8	68	M	61	Colon	Cancer adjacent normal colonic tissue	–	–	–	NAT
G9	69	M	68	Colon	Cancer adjacent normal colonic tissue	–	–	–	NAT
G10	70	F	31	Colon	Cancer adjacent normal colonic tissue	–	–	–	NAT
H1	71	M	35	Colon	Normal colonic tissue	–	–	–	Normal
H2	72	F	21	Colon	Normal colonic tissue	–	–	–	Normal
H3	73	M	40	Colon	Normal colonic tissue	–	–	–	Normal
H4	74	M	35	Colon	Normal colonic tissue	–	–	–	Normal
H5	75	M	45	Colon	Normal colonic tissue	–	–	–	Normal
H6	76	M	45	Colon	Normal colonic tissue	–	–	–	Normal
H7	77	M	32	Colon	Normal colonic tissue (smooth muscle tissue)	–	–	–	Normal
H8	78	M	30	Colon	Normal colonic tissue	–	–	–	Normal
H9	79	M	35	Colon	Normal colonic tissue	–	–	–	Normal
H10	80	M	35	Colon	Normal colonic tissue	–	–	–	Normal
–	–	M	42	Adrenal gland	Pheochromocytoma (tissue marker)	–			Malignant

Pos:Position; No.:Number are listed. Tissue microarray were purchased from US Biomax, Inc.
