# Supplementary material for: Loss of zinc‐finger protein 143 contributes to tumour progression by interleukin‐8‐CXCR axis in colon cancer
Source: J Cell Mol Med. 2019 Apr 1;23(6):4043–53. doi: 10.1111/jcmm.14290 (PMC6533486; doi:10.1111/jcmm.14290)
Supplement: Supplementary file 3 [file JCMM-23-4043-s003.pdf]

Supplementary Table S3:IHC Scoring Results

All tissue microarray stained twice and scored by professional pathologist from no staining to strong staining, which were converted into 1 to 5, and analyzed.

No staining	-	(1)
very weak staining	+	(2)
weak staining	++	(3)
Moderate staining	+++	(4)
Strong staining	++++	(5)

BS050002b														
Antibody	ZNF143	ZNF143	IL8	IL8	pSTAT3	pSTAT3								
Dilution	1/100	1/100	1/500	1/500	1/100	1/100								
Slide no.	515	515	607	607	606	606								
Date of st	08-06-201	08-06-201	11-07-201	11-07-201	11-07-201	11-07-2017	Sex	Age	Organ	Pathology diagnosis	Grade	Stage	TNM	Type
Sample	ZNF143	ZNF143	IL8	IL8	pSTAT3	pSTAT3								
A1	1	[-]	1	[-]	1	[-]	F	42	Colon	Adenocarcinoma	1	IIB		Malignant
A2	2	[+]	1	[-]	1	[-]	F	71	Colon	Adenocarcinoma	2	IIIB		Malignant
A3	1	[-]	4	[+++]	1	[-]	F	63	Colon	Adenocarcinoma	3	IIA		Malignant
A4	3	[++]	3	[++]	3	N-[++]/ Cyto [-]	M	71	Colon	Adenocarcinoma	1	IIIC		Malignant
A5	2	[+]	1	[-]	1	[-]	M	62	Colon	Adenocarcinoma	2	IV		Malignant
A6	0	No tumor	0	No tumor	0	No tumor	F	61	Colon	Adenocarcinoma	0	IIB		Malignant
A7	1	[-]	4	[+++]	1	[-]	F	46	Colon	Adenocarcinoma	2	I		Malignant
A8	1	[-]	1	[-]	1	[-]	M	61	Colon	Adenocarcinoma	3	IIB		Malignant
A9	3	[++]	3	[++]	3	N-[++]/ Cyto [-]	M	64	Colon	Adenocarcinoma	3	IIB		Malignant
A10	3	[++]	1	[-]	1	[-]	F	74	Colon	Adenocarcinoma	3	III		Malignant
B1	1	[-]	4	[+++]	1	[-]	M	33	Colon	Adenocarcinoma	3	IIA		Malignant
B2	1	[-]	2	[+]	1	[-]	M	65	Colon	Adenocarcinoma	3	III		Malignant
B3	0	No tumor	0	No tumor	0	No tumor	M	36	Colon	Adenocarcinoma	3	IIB		Malignant
B4	3	[++]	1	[-]	1	[-]	M	47	Colon	Adenocarcinoma	3	III		Malignant
B5	3	[++]	1	[-]	1	[-]	M	71	Colon	Adenocarcinoma	3	III		Malignant
B6	0	No tissue	0	No tissue	0	No tissue	M	72	Colon	Adenocarcinoma	3	IIB		Malignant
B7	2	[+]	1	[-]	1	[-]	M	75	Colon	Adenocarcinoma	3	IIB		Malignant
B8	2	[+]	1	[-]	1	[-]	F	59	Colon	Mucinous adenocarcinoma	3	III		Malignant
B9	3	[++]	4	[+++]	3	N-[++]/ Cyto [-]	F	48	Colon	Mucinous adenocarcinoma	3	IV		Malignant
B10	3	[++]	1	[-]	1	[-]	M	49	Colon	Mucinous adenocarcinoma	2	IIIB		Malignant
C1	3	[++]	3	[++]	4	N-[+++]/ Cyto [-]	F	42	Abdominal cavity	Metastatic mucinous adenocarcinoma from colon	3			MET
C2	3	[++]	1	[-]	1	[-]	M	58	Liver	Metastatic adenocarcinoma from colon	2			MET
C3	1	[-]	1	[-]	1	[-]	F	53	Mesentery	Metastatic adenocarcinoma of lymph node from colon	2			MET
C4	3	[++]	1	[-]	1	[-]	M	78	Pancreas	Metastatic adenocarcinoma of lymph node from colon	2			MET
C5	0	No tissue	0	No tissue	0	No tissue	M	71	Lymph node	Metastatic adenocarcinoma from colon of No.4	2			MET
C6	2	[+]	1	[-]	1	[-]	M	55	Lymph node	Metastatic adenocarcinoma from colon	2			MET
C7	0	No tumor	0	No tumor	0	No tumor	M	65	Lymph node	Metastatic adenocarcinoma from colon	2			MET
C8	3	[++]	1	[-]	3	N-[++]/ Cyto [-]	M	30	Lymph node	Metastatic mucinous adenocarcinoma from colon	3			MET
C9	3	[++]	1	[-]	1	[-]	F	57	Lymph node	Metastatic adenocarcinoma from colon with necrosis	2			MET
C10	2	[+]	3	[++]	1	[-]	F	51	Lymph node	Metastatic adenocarcinoma from colon	3			MET
D1	0	No tumor	0	No tumor	0	No tumor	F	74	Lymph node	Metastatic adenocarcinoma from colon	2			MET
D2	0	No tumor	0	No tumor	0	No tumor	F	57	Lymph node	Metastatic adenocarcinoma from colon (tumor necrosis)				MET
D3	4	[+++]	1	[-]	1	[-]	M	58	Lymph node	Metastatic adenocarcinoma from colon				MET
D4	0	No tumor	0	No tumor	0	No tumor	M	62	Lymph node	Metastatic adenocarcinoma from colon				MET
D5	1	[-]	1	[-]	1	[-]	M	48	Lymph node	Metastatic adenocarcinoma from colon				MET
D6	2	[+]	2	[+]	1	[-]	F	68	Lymph node	Metastatic adenocarcinoma from colon				MET
D7	2	[+]	1	[-]	1	[-]	M	70	Lymph node	Metastatic adenocarcinoma from colon				MET
D8	2	[+]	1	[-]	1	[-]	F	72	Lymph node	Metastatic mucinous adenocarcinoma from colon				MET
D9	0	No tumor	0	No tumor	0	No tumor	M	58	Lymph node	Metastatic adenocarcinoma from colon				MET
D10	0	No tumor	0	No tumor	0	No tumor	F	38	Lymph node	Metastatic signet ring cell carcinoma from colon				MET
E1	3	[++]	3	[++]	1	[-]	F	48	Colon	Canalicular adenoma				
E2	2	[+]	3	[++]	1	[-]	F	67	Colon	Tubulovillous adenoma				Benign
E3	1	[-]	4	[+++]	1	[-]	F	66	Colon	Tubulovillous adenoma				Benign
E4	2	[+]	3	[++]	1	[-]	M	35	Colon	Tubulovillous adenoma				Benign
E5	3	[++]	3	[++]	1	[-]	F	50	Colon	Tubulovillous adenoma				Benign
E6	3	[++]	4	[+++]	2	[+]	M	55	Colon	Hyperplastic polyp				Polyp
E7	1	[-]	1	[-]	1	[-]	F	58	Colon	Adenomatous polyp				Polyp
E8	0	No tissues	0	No tissues	0	No tissues	M	41	Colon	Adenomatous polyp				Polyp
E9	4	[+++]	0	No tumor[	3	No tumorN-[++]/	M	45	Colon	Hyperplastic polyp				Polyp
E10	2	[+]	3	[++]	1	[-]	F	20	Colon	Polyp				Polyp
F1	3	[++]	3	[++]	1	[-]			Colon	Crohn's disease (chronic colitis)				Inflammation
F2	0	No tumor	0	No tumor	1	[-]			Colon	Crohn's disease (chronic colitis)				Inflammation
F3	2	[+]	1	[-]	1	[-]			Colon	Crohn's disease (chronic colitis)				Inflammation
F4	2	[+]	4	[+++]	1	[-]			Colon	Crohn's disease				Inflammation
F5	1	[-]	3	[++]	1	[-]			Colon	Tuberculosis (fibrous tissue and smooth muscle)				TB
F6	3	[++]	3	[++]	1	[-]			Colon	Chronic colitis				Inflammation
F7	3	[++]	3	[++]	1	[-]			Colon	Chronic colitis				Inflammation
F8	0	No tissue	0	No tissue	0	No tissue			Colon	Chronic colitis				Inflammation
F9	4	[+++]	3	[++]	1	[-]			Colon	Chronic colitis				Inflammation
F10	3	[++]	3	[++]	1	[-]			Colon	Chronic colitis				Inflammation
G1	3	[++]	1	[-]	3	N-[++]/ Cyto [-]			Colon	Cancer adjacent normal colonic tissue				NAT
G2	3	[++]	4	[+++]	2	N-[-]/ Cyto [+]			Colon	Cancer adjacent normal colonic tissue				NAT
G3	2	[+]	1	[-]	1	[-]			Colon	Cancer adjacent normal colonic tissue				NAT
G4	3	[++]	1	[-]	1	[-]			Colon	Cancer adjacent normal colonic tissue				NAT
G5	2	[+]	3	[++]	1	[-]			Colon	Cancer adjacent normal colonic tissue				NAT
G6	2	[+]	4	[+++]	1	[-]			Colon	Cancer adjacent normal colonic tissue				NAT
G7	3	[++]	1	[-]	1	[-]			Colon	Cancer adjacent normal colonic tissue				NAT
G8	0	No tumor	0	No tumor	0	No tumor			Colon	Cancer adjacent normal colonic tissue				NAT
G9	1	[-]	3	[++]	1	[-]			Colon	Cancer adjacent normal colonic tissue				NAT
G10	2	[+]	3	[++]	1	[-]			Colon	Cancer adjacent normal colonic tissue				NAT
H1	3	[++]	1	[-]	1	[-]			Colon	Normal colonic tissue				Normal
H2	1	[-]	1	[-]	1	[-]			Colon	Normal colonic tissue				Normal
H3	3	[++]	1	[-]	1	[-]			Colon	Normal colonic tissue				Normal
H4	3	[++]	1	[-]	1	[-]			Colon	Normal colonic tissue				Normal
H5	3	[++]	1	[-]	1	[-]			Colon	Normal colonic tissue				Normal
H6	0	No tumor	0	No tumor	0	No tumor			Colon	Normal colonic tissue				Normal
H7	0	No tumor	0	No tumor	0	No tumor			Colon	Normal colonic tissue				Normal
H8	3	[++]	1	[-]	1	[-]			Colon	Normal colonic tissue				Normal
H9	4	[+++]	1	[-]	1	[-]			Colon	Normal colonic tissue				Normal
H10	3	[++]	1	[-]	1	[-]			Colon	Normal colonic tissue				Normal
